# Supplementary material for: Non-orthogonal optical multiplexing empowered by deep learning
Source: Nat Commun. 2024 Feb 21;15:1580. doi: 10.1038/s41467-024-45845-4 (PMC10881499; doi:10.1038/s41467-024-45845-4)
Supplement: Supplementary file 1 — Supplementary Information [file 41467_2024_45845_MOESM1_ESM.pdf]

Supplementary Information for Non-orthogonal optical multiplexing empowered by deep learning

Tuqiang Pan[1,2,3], Jianwei Ye[1,2,3], Haotian Liu[1,2], Fan Zhang[1,2], Pengbai Xu[1,2], Ou Xu[1,2], Yi Xu[1,2,4*], and Yuwen Qin[1,2,4*]

[1]Key Laboratory of Photonic Technology for Integrated Sensing and Communication, Ministry of Education, Guangzhou, 510006, China
[2]Guangdong Provincial Key Laboratory of Information Photonics Technology, Institute of Advanced Photonic Technology, School of Information Engineering, Guangdong University of Technology, Guangzhou, 510006, China
[3]These authors contributed equally: Tuqiang Pan, Jianwei Ye
[4]These authors jointly supervised this work: Yi Xu, Yuwen Qin
[*]yixu@gdut.edu.cn, qinyw@gdut.edu.cn

Supplementary Note 1 — Performance of the non-orthogonal multiplexing using the SLRnet

Compared with PCC, structure similarity index measure (SSIM) is an alternative quantitative measurement which aligns with the non-linear perception of human eyes. As shown in Supplementary Figure 1, the evolutions of the corresponding retrieved SSIM for the Fig. 3 of main text are larger than 0.90 after 100 epochs.

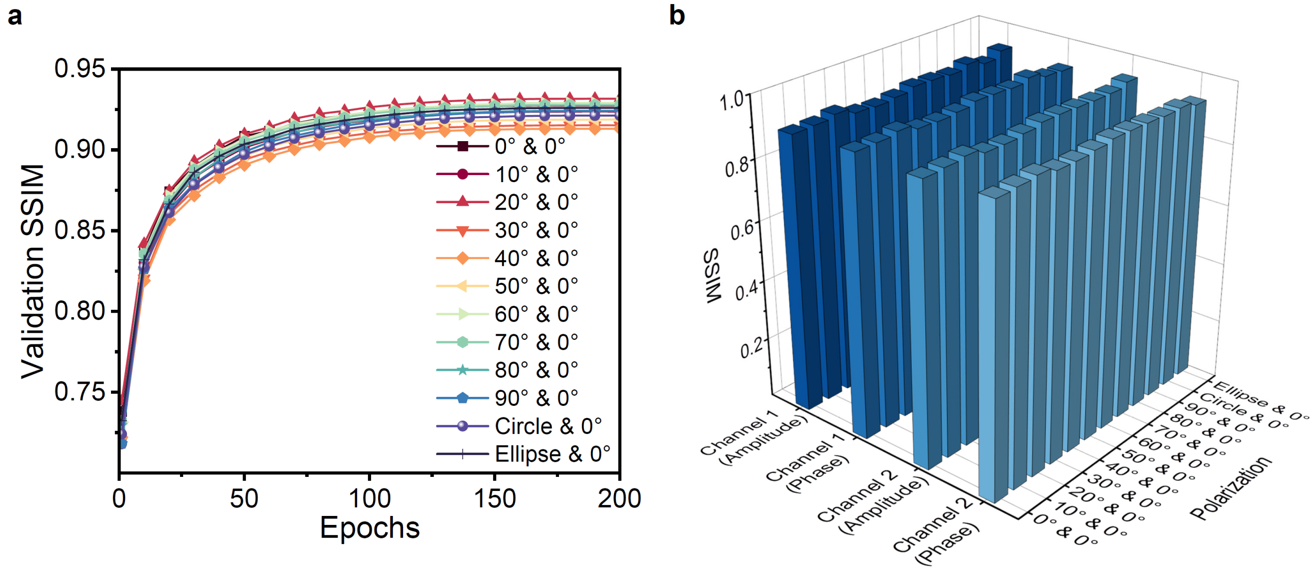

Supplementary Figure 1. Performance of the non-orthogonal multiplexing using the SLRnet. a Averaged SSIMs of the validation dataset during training procedures, where multiplexing scenarios of two input channels over a 1 m MMF with arbitrary polarization combinations are shown. The angles of polarizations with respect to the horizon line are indicated. Here, circle and ellipse indicate circular and elliptical polarizations, respectively. **b** The SSIMs of retrieved information in different multiplexing channels at the final epoch. SSIM: Structure Similarity Index Measure

Supplementary Note 2 — Results for more complex information encoded in the non-orthogonal input channels

The results considering more complex grayscale encoded information from the CelebA face dataset[1] are shown in Supplementary Figure 2. These results validate that the SLRnet has an excellent ability to retrieve complicate multiplexing inputs encoded in the non-orthogonal channels. The average SSIM and PCC of the whole validation set are 0.741 and 0.927, respectively. The non-orthogonal multiplexing of uncorrelated random binary data through an MMF is further demonstrated, as shown in Supplementary Figure 3a. The averaged bit accuracy rate is about 98%, validating the potential of applying the reported non-orthogonal optical multiplexing concept for binary data transmission. It should be pointed out that when the multiplexing capacity is increased, the bit accuracy rate is decreased with a fix amount of dataset, as shown by Supplementary Figure 3b. It indicates a challenge of the proposed approach, where increasing the resolution of input multiplexing channels will require exponentially increasing amounts of data.

The non-orthogonal multiplexing of general natural scene images from ImageNet database[2] is also demonstrated, as shown in Fig. 6a of the main text. The achieved averaged SSIM/PCC is 0.737/0.905, which are comparable to the results of the orthogonal counterpart using physically-informed approach for transmitting natural scene images. Because the amplitude and phase modulations are applied using a phase-only SLM, the modulation precision is limited. If we only encode the information in the phase dimension, the achieved averaged fidelity can be up to 0.819/0.945 (SSIM/PCC), which is substantially improved compared with the complex modulation case. Typical results for the non-orthogonal multiplexing of general natural scene images from ImageNet database[2] and snapshots from Muybridge recordings are shown in Fig. 6b and c of the main text, respectively. It should be emphasized that the data from the Muybridge recordings (see Fig. 6c) is not belong to the same type of training dataset of the neural network.

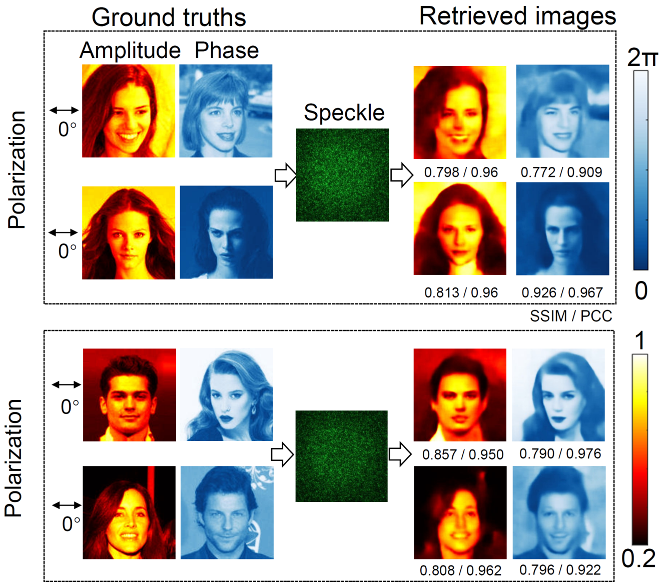

Supplementary Figure 2. Results of non-orthogonal multiplexing over a 1 m MMF. The ground truths, the speckle output and the corresponding retrieved light field information by the SLRnet for more complicate input information are shown, where their corresponding SSIM and PCC are given, respectively. Colorbars are also provided for the grayscale images encoded in the amplitude and phase of light field. These images are from the CelebA dataset[1].

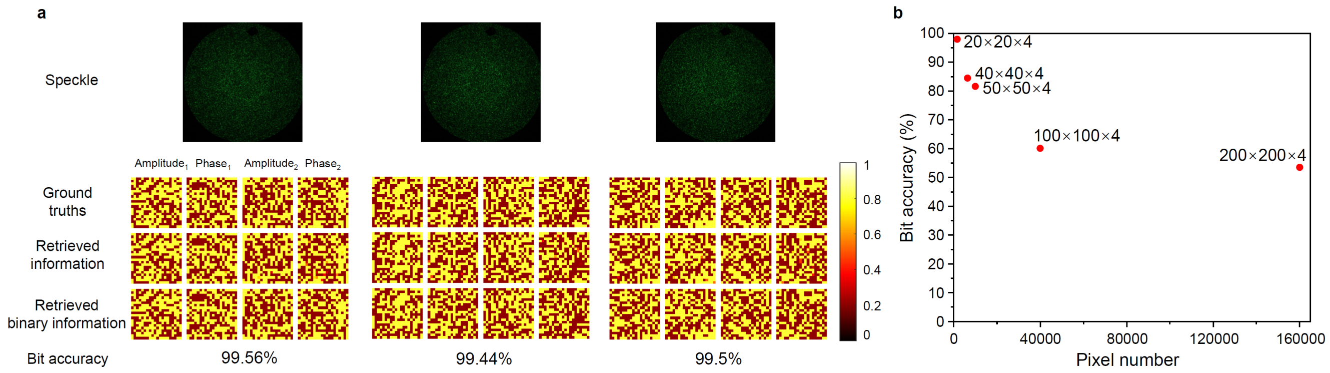

Supplementary Figure 3. Demultiplexing results for the non-orthogonal optical multiplexing of binary random binary data in a 1m MMF. a The speckle outputs, the corresponding ground truths, the retrieved results of neural network, the binarized results and the bit accuracy rate are given, respectively. The length of the MMF is 1m. It should be pointed out that the SLRnet here is revised to facilitate the demultiplexing of the random binary data, as elaborated in our new Supplementary Note 7. The amplitude and phase are self-normalized. The ground truths of uncorrelated random binary data are generated by the uniform random function of Matlab, which consists of 20 x 20 pixels. These results are on the test set. **b** The dependence of bit accuracy rate on the input pixel number. The total input pixel number can be calculated by resolutions × 2(amplitude and phase) × 2 (beam number), as indicated in the inset. The sizes of all datasets are fixed at 30,000 pairs.

Supplementary Note 3 — Comparison of performance between orthogonal and non-orthogonal optical multiplexing

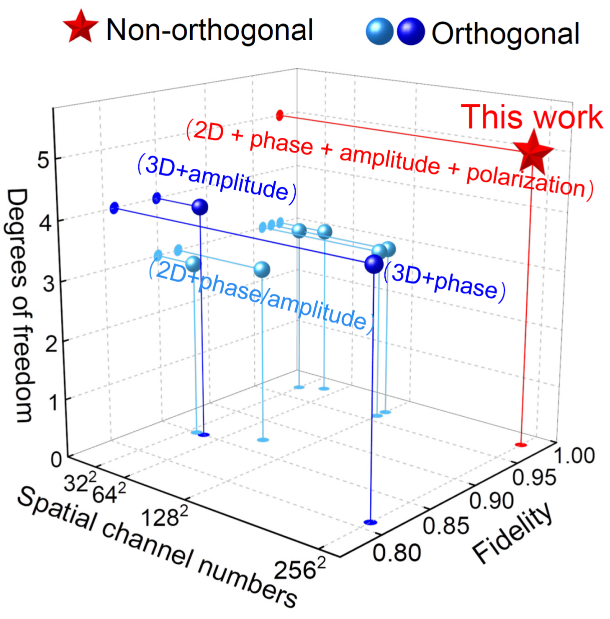

Supplementary Figure 4. Comparison between non-orthogonal optical multiplexing and its orthogonal counterparts. Typical works using deep learning to transmit multiplexed information over a multimode fiber. The corresponding optical degrees of freedom, fidelity and spatial channel numbers involved in multiplexing for these works are provided, respectively. Here, 2D and 3D indicate two and three spatial dimensions, respectively. The pentagram represents multiplexing using non-orthogonal channels, while spheres represent the orthogonal counterparts.

To date, up to four optical degrees of freedom have been used for orthogonal optical multiplexing in multimode fibers[3,4], where information multiplexed in different combinations of three-dimensional space (X, Y, and Z), amplitude and phase encoded in the input wavefront have been achieved by utilizing the deep learning methods[3–10]. The results for orthogonal optical multiplexing are summarized in Supplementary Figure 4, where their corresponding optical degrees of freedom, spatial channel numbers and retrieved fidelity are shown, respectively.

Supplementary Note 4 — Monitoring of experimental variation

To monitor the perturbation to the MMF from environment, the PCC evaluating the correlation between an instantaneous speckle pattern (every 100 frames) with the first one is calculated, as shown in Supplementary Figure 5. The non-orthogonal multiplexing case of uncorrelated random binary data shown in the Supplementary Figure 3 is considered. The incident light field is fixed during the measurement. As can be seen from this figure, both the training and validation datasets contain the perturbation from the environment. Therefore, the trained SLRnet possesses certain robustness against the perturbation from the environment.

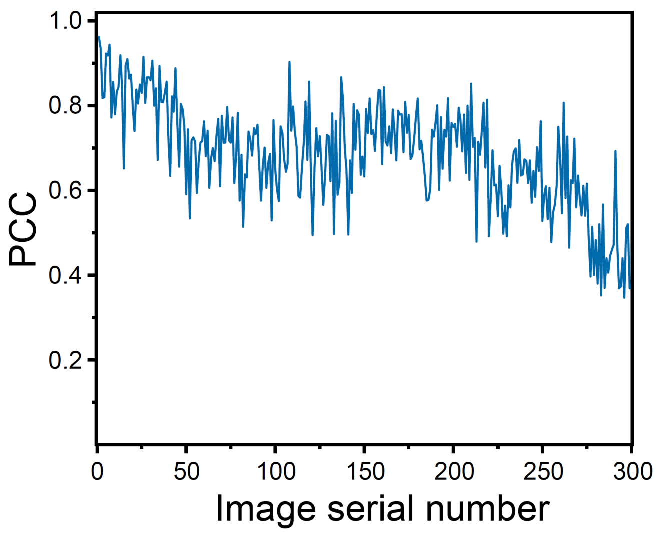

Supplementary Figure 5. The stability monitoring of the MMF under a fixed input wavefront. The PCC evaluating the correlation between an instantaneous speckle pattern (every 100 frames) with the first one is presented.

Supplementary Note 5 — Experimental setup

The experimental setup is shown in Supplementary Figure 6. The details of experimental parameters can be found in the Methods section of the main text. Different polarization combinations were achieved by rotating a half-wave plate or a quarter-wave plate.

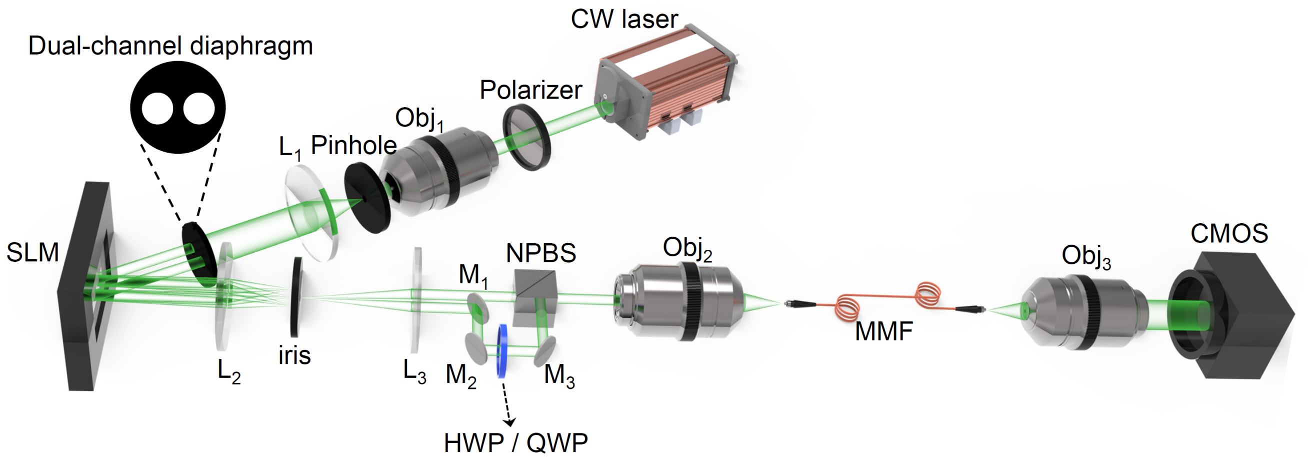

Supplementary Figure 6. The experimental setup for non-orthogonal optical multiplexing empowered by deep learning. Obj : Objective lens (Obj_1 : 20×, NA = 0.4; Obj_2 and Obj_3 : 10×, NA = 0.25). L : Lens (L_1, L_2 and L_3 : f = 150 mm). M : Mirror. NPBS : Non-polarizing beam splitter. MMF : Multimode fiber. HWP : Half-wave plate. QWP : Quarter-wave plate.

Supplementary Note 6 — The sizes of all used datasets and their corresponding fidelity

Data type	Resolution	Modulation scheme	Size	Correspondence	Averaged fidelity (SSIM/PCC or Bit accuracy)
Fashion-MNIST	256 × 256	Amplitude & Phase	30000	Figure 4 & Figure 5	0.93 / 0.98
CelebA face	256 × 256	Amplitude & Phase	34000	Figure S2	0.741 / 0.927
Random binary data	20 × 20	Amplitude & Phase	30000	Figure S3	98%
ImageNet	128 × 128	Amplitude & Phase	50000	Not show	0.706 / 0.888
ImageNet	128 × 128	Pure phase	50000	Figure 6b &Figure 6c	0.819 / 0.945
ImageNet	128 × 128	Amplitude & Phase	80000	Figure 6a	0.737 / 0.905

Supplementary Table 1. Summaries of the parameters for all used datasets Data type, resolution, modulation scheme, size of dataset, correspondence and averaged fidelity. All validation sets are 10% of their corresponding datasets.

Supplementary Note 7 — The architectures of each module in the SLRnet

The FC module consists of a fully connected (FC) layer and an adaptive average pooling layer, where the input of the FC layer (via flatten) is a speckle image with 200 × 200 pixels, as shown in Supplementary Figure 7. And the output is a feature map with 128 × 128 pixels (via unflatten), which is up sampled to 256 × 256 pixels via an adaptive average pooling layer. For training using the ImageNet database, the adaptive average pooling layer is not

used. There are two versions of ResConv, where the first version is shown in the blue box outlined by dashed lines. In order to match the output feature map from the FC module, the number of its input channel is only 1. And the stride of the convolution layer is set to 1. In the first ResConv, downsampling is not executed. The second version of ResConv is shown in the blue box outlined by solid lines. When its input channel number is C, its output channel is 2C. At the same time, the stride of the convolution layer is set to 2, where the size of the output feature map becomes half of the input. There are four convolution layers in ResConvT, one of which is a transposed convolution layer with a stride of 2, which realizes upsampling of the input feature map with learning. This is different from the bilinear interpolation algorithm used in Unet. The feature map through ResConvT compresses the channel number to one-third of its original size.

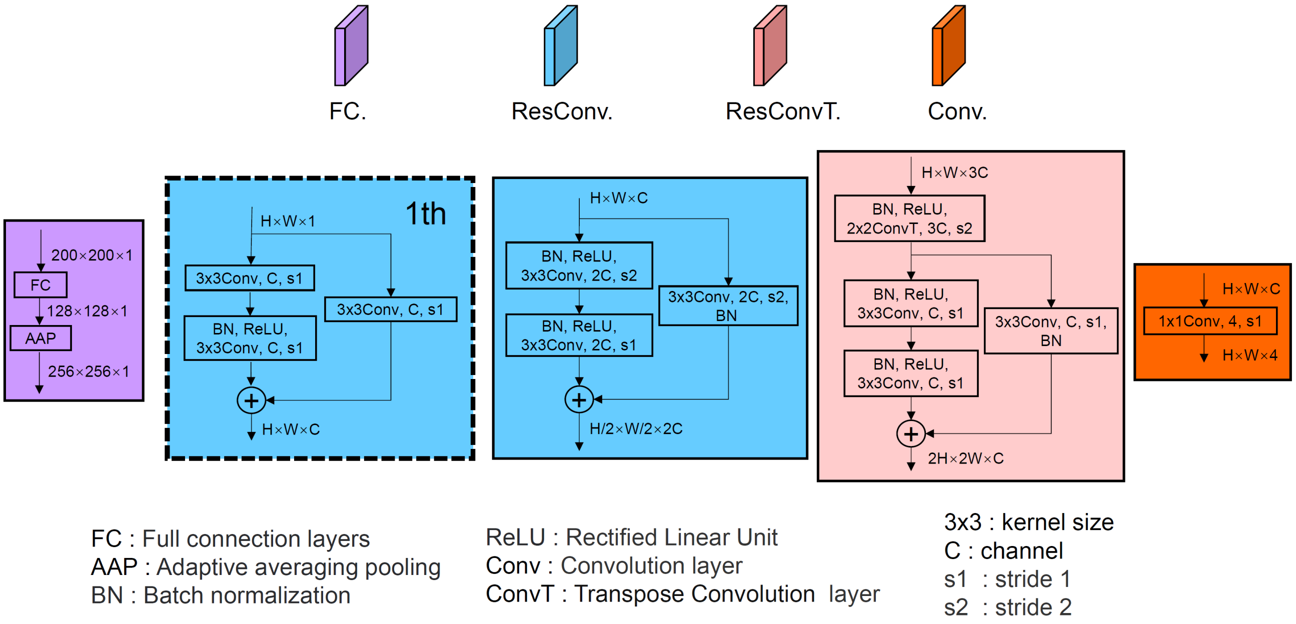

Supplementary Figure 7. The detailed architectures of each module in the SLRnet. Here, the downsampling of ResConv is implemented using a convolutional layer with a stripe of 2 instead of a pooling layer. The upsampling of ResConv is implemented using a transposed convolutional layer with a stripe of 2 instead of an interpolation algorithm. An adaptive pooling layer is used in the FC module to match the output size.

In order to facilitate the decoding of uncorrelated random binary data, we make minor changes to the SLRnet, where the revised part of network is shown in Supplementary Figure 8. The modified SLRnet uses a ResConvD module instead of the ResConvT module, and eliminates skip connections in the ResUnet. In that case, SLRnet does not perform upsampling after downsampling to a certain size.

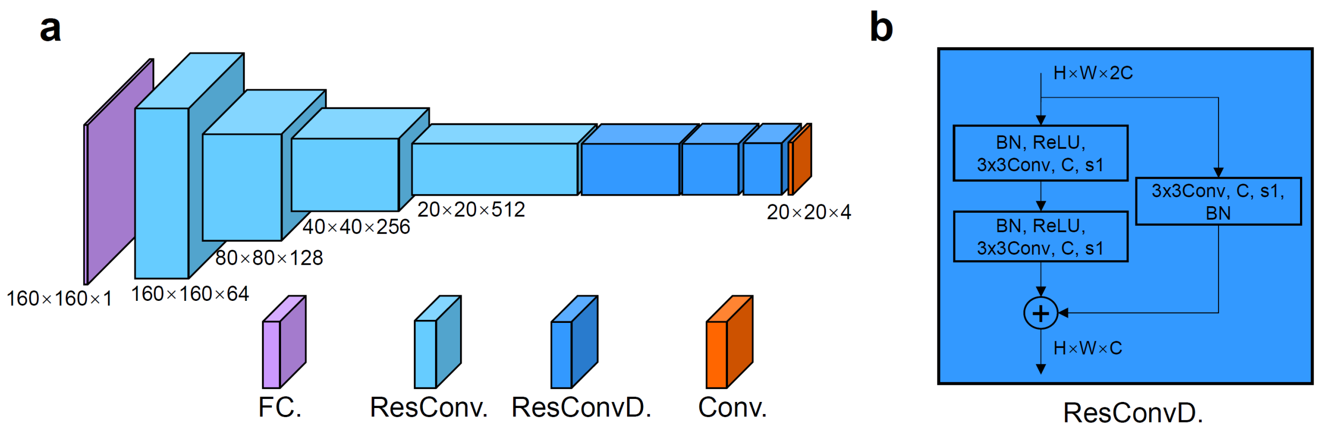

Supplementary Figure 8. The modified SLRnet for the non-orthogonal multiplexing of random binary data. a It consists of the same base modules, where skipping connections are eliminated. ResConvT is replaced by ResConvD. **b** The configuration of ResConvD.

Supplementary Note 8 — Learning rate adjustment strategy

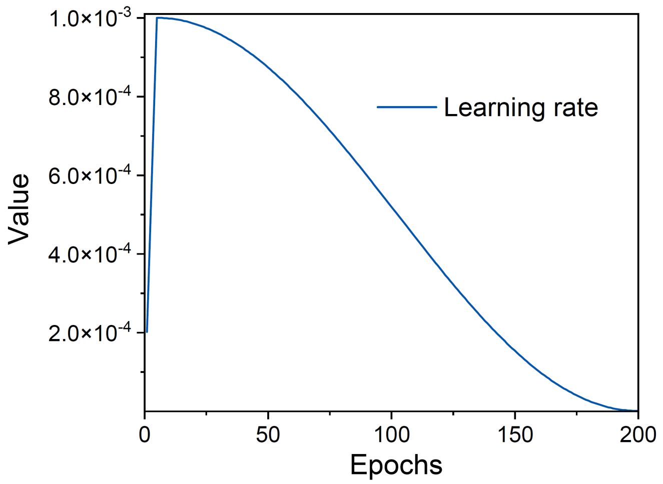

Supplementary Figure 9. Adjustment of learning rate during different epochs.

The initial learning rate is set at 2×10^{-4}, rising to 1×10^{-3} after five epochs of warm-up, and subsequently dropping to 0 at the last epoch according to the cosine annealing schedule[11], as shown Supplementary in Figure 9.

References

1. Zhang, Y. *et al.* Celeba-spoof: Large-scale face anti-spoofing dataset with rich annotations. In *Computer Vision–ECCV 2020: 16th European Conference, Glasgow, UK, August 23–28, 2020, Proceedings, Part XII 16*, 70–85 (Springer, 2020).

2. Deng, J. *et al.* Imagenet: A large-scale hierarchical image database. In *2009 IEEE Conference on Computer Vision and Pattern Recognition*, 248–255 (2009).

3. Fan, W. *et al.* Single-shot recognition of 3D phase images with deep learning. *Laser Photonics Rev.* **16**, 2100719 (2022).

4. Wang, L. *et al.* High-speed all-fiber micro-imaging with large depth of field. *Laser Photonics Rev.* **16**, 2100724 (2022).

5. Borhani, N., Kakkava, E., Moser, C. & Psaltis, D. Learning to see through multimode fibers. *Optica* **5**, 960–966 (2018).

6. Rahmani, B., Loterie, D., Konstantinou, G., Psaltis, D. & Moser, C. Multimode optical fiber transmission with a deep learning network. *Light. Sci. Appl.* **7**, 69 (2018).

7. Caramazza, P., Moran, O., Murray-Smith, R. & Faccio, D. Transmission of natural scene images through a multimode fibre. *Nat. Commun.* **10**, 2029 (2019).

8. Resisi, S., Popoff, S. M. & Bromberg, Y. Image transmission through a dynamically perturbed multimode fiber by deep learning. *Laser Photonics Rev.* **15**, 2000553 (2021).

9. Liu, Z. *et al.* All-fiber high-speed image detection enabled by deep learning. *Nat. Commun.* **13**, 1433 (2022).

10. Tang, P. *et al.* Learning to transmit images through optical speckle of a multimode fiber with high fidelity. *Appl. Phys. Lett.* **121**, 081107 (2022).

11. Liu, Z. *et al.* A ConvNet for the 2020s. In *Proceedings of the IEEE/CVF Conference on Computer Vision and Pattern Recognition (CVPR)*, 11976–11986 (2022).
